# Editorial: Molecular Links Between Mitochondrial Damage and Parkinson's Disease and Related Disorders

**DOI:** 10.3389/fcell.2021.734475

**Published:** 2021-08-06

**Authors:** Yuzuru Imai, Kiyoung Kim, Zhihao Wu, Shigeto Sato

**Affiliations:** ^1^Department of Neurology, Juntendo University Graduate School of Medicine, Tokyo, Japan; ^2^Department of Research for Parkinson's Disease, Juntendo University Graduate School of Medicine, Tokyo, Japan; ^3^Department of Medical Biotechnology, Soonchunhynag University, Asan, South Korea; ^4^Department of Medical Sciences, Soonchunhynag University, Asan, South Korea; ^5^Department of Biological Sciences, Southern Methodist University, Dallas, TX, United States

**Keywords:** Ca^2+^, astrocyte, microglia, STING, Parkin (PARK2), USP14, peroxiredoxin, NADPH oxidase

Mitochondria are organelles that play a variety of roles, including energy production, regulation of intracellular Ca^2+^, lipid and iron metabolism, redox regulation, inflammation, and cell death. The involvement of mitochondria in the etiology of Parkinson's disease has been long suspected; however, the reason for the selective degeneration of dopaminergic neurons remains a mystery. This Research Topic was envisioned to provide insights that could aid in uncovering this mystery and consists of seven excellent reviews, two unique perspectives, and two original papers that pioneer new fields of study. These studies demonstrate that mitochondrial dysfunction is more complex than previously thought and its association with other organelles as well as the association between neurons and glia must be elucidated.

Jung et al. provide a concise overview of recent studies on mitochondrial Ca^2+^ regulation during neural activity and mechanisms of brain aging and neurodegeneration caused by functional abnormalities in molecules involved in mitochondrial Ca^2+^ regulation. In particular, Table 1 provides an excellent summary of studies that involve genetically encoded calcium indicators and will be of great help to researchers who will be conducting studies in this field.

A review by Lin et al. indicates the importance of communication between mitochondria and other organelles, such as the endoplasmic reticulum, the Golgi apparatus, peroxisomes, and lysosomes. At organelle contacts, Ca^2+^, lipids, proteins, and other substances are exchanged. This area deserves further attention as VPS13, the protein family responsible for neurodegenerative diseases, including Parkinson's disease, functions at the level of these organelles (Ugur et al., 2020). This review describes findings and genetic evidence that supports the theory of organelle communication disorders leading to neurodegeneration.

Nicoletti et al. present an extensive list of studies reporting the effects of Parkinson's disease-related gene mutations on the mitochondria. In addition, they mention the pathogenic roles of mitochondria in atypical parkinsonism, progressive supranuclear palsy (in which tau aggregates accumulate in both neurons and astrocytes), and Huntington's disease (in which neurotoxic protein aggregation is caused by N-terminal polyglutamine elongation of Huntingtin protein). Moreover, Li et al. briefly summarize the effects of 11 monogenic Parkinson's disease genes (*SNCA, PRKN, PINK1, DJ-1, LRRK2, ATP13A2, PLA2G6, FBXO6, VPS35, CHCHD2, and VPS13C*) on mitochondrial function and α-synuclein aggregation. However, further discussion is needed to decipher whether mitochondrial abnormalities are the cause or the consequence of these diseases.

The involvement of astrocytes in the pathogenesis of Parkinson's disease is of recent interest. Bantle et al. summarize Research Topics related to astrocyte mitochondria, such as metabolism of glutamate, Ca^2+^, and lipids as well as inflammation. Mitochondrial transfer between astrocytes and neurons is a newly discovered phenomenon and a topic of peculiar interest, including its therapeutic potential (Hayakawa et al., 2016). Similarly, Jeon et al. discuss a different perspective on glial mitochondrial function and α-synuclein toxicity. Their review discusses the potential modulation of α-synuclein propagation to neurons and oligodendrocytes by glial mitochondrial dysfunction and neuroinflammation.

Mitochondrial dysfunction is hallmark of cancer, leading to an increase in glycolytic metabolism, which is known as the Warburg effect. Lu and Ho have proposed a similar scenario that occurs in brain tumors where tumor-associated macrophages and microglia play an active role in cancer immunity. Mitochondrial dysfunction induces mtDNA release, which in turn activates glia-associated macrophages and microglia to elicit anti-tumor response through the STING signaling (Mathur et al., 2017). They indicate that Parkinson's disease-associated PINK1-Parkin signaling is a potential therapeutic target as it negatively regulates the STING pathway through the removal of damaged mitochondria (Sliter et al., 2018). Furthermore, PINK1-Parkin signaling is also believed to be involved in mitochondrial quality control *via* mitochondrial motility arrest and mitophagy (Imai, 2020). In contrast, ubiquitin-specific proteases, which cleave polyubiquitin chains, possess the potential to suppress the progression of mitophagy (Bingol et al., 2014; Niu et al., 2020). Among them, USP14 is involved in both the ubiquitin-proteasome system (Lee et al., 2010; Kim and Goldberg, 2017) and mitophagy (Chakraborty et al., 2018). The study by Banerjee et al. on the expression of USP14 and Prohibitin 2, an inner mitochondrial membrane LC3 receptor (Wei et al., 2017), in a variety of brain regions of both young and adult rats discusses the potential of USP14 as a therapeutic target.

Odnokoz et al. report that the inhibition of mitochondrial thiol-dependent peroxidases, i.e., peroxiredoxins (Prxs), in a *Drosophila* model leads to a significant reduction in *Drosophila* lifespan. Although the physiological functions of Prxs remain to be elucidated, it has been suggested that Prxs may play a role in suppressing premature aging. The elucidation of its effects on neurodegenerative models is warranted in the future.

Liu et al. reveal that the combination of NADPH, which is required for the production of reduced glutathione, and NADPH oxidase (NOX) inhibitors effectively suppresses kainic acid-induced neurotoxicity. As NOX produces reactive oxygen species with NADPH (Bedard and Krause, 2007), inhibition of NOX in turn inhibits the production of reactive oxygen species. Although there are still several issues to be addressed before NADPH can be used clinically, this study provides a possible therapeutic approach.

Prasuhn et al. discuss, in detail, mitochondria as a prospective therapeutic target. In particular, brain imaging analysis at the prodromal stage and inexpensive exercise therapy seem to be realistic methods for early detection and control of mitochondrial diseases, respectively. Moreover, they point out that clinical phenotypes of hereditary mitochondrial diseases do not usually have attributes similar to those of Parkinson's disease or related disorders. Therefore, focusing solely on the well-known functions of mitochondria may hinder in the elucidation of the real causes of neurodegeneration.

The wide variety of reviews and original publications listed here, which address the multifaceted roles of mitochondria and their communication with other organelles within and beyond the cell, confirm that mitochondrial studies remain a research focus in the field of neurodegenerative diseases. Furthermore, the studies listed here will serve as a valuable introduction to this field.

## Author Contributions

YI wrote the manuscript. YI, KK, ZW, and SS corrected the manuscript. All authors contributed to the article and approved the submitted version.

## Conflict of Interest

The authors declare that the research was conducted in the absence of any commercial or financial relationships that could be construed as a potential conflict of interest.

## Publisher's Note

All claims expressed in this article are solely those of the authors and do not necessarily represent those of their affiliated organizations, or those of the publisher, the editors and the reviewers. Any product that may be evaluated in this article, or claim that may be made by its manufacturer, is not guaranteed or endorsed by the publisher.
